# Immune Checkpoint Inhibitor-Associated Colitis: From Mechanism to Management

**DOI:** 10.3389/fimmu.2021.800879

**Published:** 2021-12-21

**Authors:** Liansha Tang, Jialing Wang, Nan Lin, Yuwen Zhou, Wenbo He, Jiyan Liu, Xuelei Ma

**Affiliations:** ^1^ Department of Biotherapy, Cancer Center, West China Hospital, Sichuan University, Chengdu, China; ^2^ Department of Neurosurgery, West China Hospital, Sichuan University, Chengdu, China

**Keywords:** immune checkpoint inhibitor, colitis, diarrhoea, mechanism, diagnosis, management, prognosis

## Abstract

Immune checkpoint inhibitors (ICIs), as one of the innovative types of immunotherapies, including programmed cell death-1 (PD-1), programmed cell death-ligand 1 (PD-L1), and cytotoxic T lymphocyte antigen 4 (CTLA-4) inhibitors, have obtained unprecedented benefit in multiple malignancies. However, the immune response activation in the body organs could arise immune-related adverse events (irAEs). Checkpoint inhibitor colitis (CIC) is the most widely reported irAEs. However, some obscure problems, such as the mechanism concerning gut microbiota, the confusing differential diagnosis with inflammatory bowel disease (IBD), the optimal steroid schedule, the reintroduction of ICIs, and the controversial prognosis features, influence the deep understanding and precise diagnosis and management of CIC. Herein, we based on these problems and comprehensively summarized the relevant studies of CIC in patients with NSCLC, further discussing the future research direction of this specific pattern of irAEs.

## Highlights

- Gut microbiota, including *Faecalibacterium prausnitzii*, *Bacteroides fragilis*, and *Lactobacillus reuteri*, plays a critical role in the development of immune checkpoint-related colitis.
- Endoscopic biopsy is the gold standard of diagnosis, while endoscopic findings were not significantly associated with the severity of immune checkpoint-related colitis.
- Current management is based on symptomatic and steroid therapy. The utilization of biological agents according to the severity of immune checkpoint-related colitis grade is reasonable and recommended.
- Longer survival was found in patients who had a deteriorated or recurrent CIC instead of improved or resolved CIC. The influence of CIC outcome on overall survival requires to be explored further.

## Introduction

Lung cancer is the leading cause of death among all cancers. In 2021, an estimated 131,880 deaths from lung cancer were recorded in the United States, with the death rate at 22% (1). As the most common type of lung cancer, non-small cell lung cancer (NSCLC) faces difficulties that over 50% of patients are diagnosed with advanced disease initially, for which the 5-year survival is nearly 5% (2). Moreover, the recommended standard treatment, chemotherapy and/or radiotherapy, offers generally modest efficacy for metastatic NSCLC patients. The 5-year survival varies from 6% to 30% when patients are treated with chemotherapy and/or radiotherapy (3, 4). Besides, target therapies, such as EGFR, ALK, and ROS1 tyrosine kinase inhibitors (TKIs), have contributed to unprecedented improvements for survival in NSCLC patients, with more moderate toxicity response than chemotherapy (5).

Recently, the emergence of immunotherapy fosters hopes for the treatment of multiple malignancies. Immune checkpoint inhibition, one of the innovative classes of immunotherapy, serves to restrain suppressive T-cell co-stimulatory signals, primarily cytotoxic CD8^+^ T cells, by inhibiting programmed death protein-1 (PD-1), PD-ligand 1 (PD-L1), and cytotoxic T-lymphocyte associated protein-4 (CTLA-4) (6, 7). PD-1 inherently presents in T cells, B cells, tumor-infiltrating lymphocytes, monocytes, and dendritic cells (DCs). PD-L1 exists on the surface of both antigen-presenting cells (APCs) and tumor cells (8–10), resulting in self-tolerance promotion and autoimmunity attenuation after interacting with PD-1. Similarly, CTLA-4 is typically expressed on CD4^+^/CD8^+^ T cells, B-cell subsets, and thymocytes, which is associated with the suppression of T-cell activity (11). Immune checkpoint inhibitors (ICIs) have the ability of strengthening the immune attack and promoting tumor killing (12, 13), thus improving the survival of cancer patients.

To date, PD-1 inhibitors (nivolumab, pembrolizumab), PD-L1 inhibitors (atezolizumab, durvalumab), and CTLA-4 inhibitor (ipilimumab) have been approved by the U.S. Food and Drug Administration (FDA) and the European Medicines Agency (EMA) for the treatment of advanced NSCLC (14). The combination of nivolumab and ipilimumab contributed to prolonged overall survival (OS) and progression-free survival (PFS) in patients with NSCLC (15–17).

However, the eliminated immunoregulatory control could cause unrestrained immune response activation in body organs (18), like the gastrointestinal tract, liver, lung, heart, skin, and arthrosis, which leads to immune-related adverse events (irAEs) ultimately (19, 20). Among clinically reported irAEs, gastrointestinal toxicity is the most common irAEs that results in ICI discontinuation (21–23). Because of the similar symptoms with inflammatory bowel disease (IBD) and/or intestinal infection, the diagnosis of checkpoint inhibitor colitis (CIC) might be confused. As a result, precise diagnosis and optimized therapy are essential for recognizing this specific pattern of irAEs.

Prior studies have demonstrated the incidence, risk factors, diagnosis, and management of CIC (24–29). Nonetheless, they have variable concentrations and orientations, which are complementary but not deep and comprehensive. This review comprehensively summarized the relevant research studies and advanced progress of ICI-related colitis, especially the aspects less mentioned before, like the mechanism, evidence-based management schedules, rechallenge of ICIs, and prognosis of CIC. Besides, we provided a summary of cases and case series regarding CIC in NSCLC, aiming at identifying the clinicopathological and endoscopic features of these typical patients.

## Incidence and Onset

**Figure 1:**
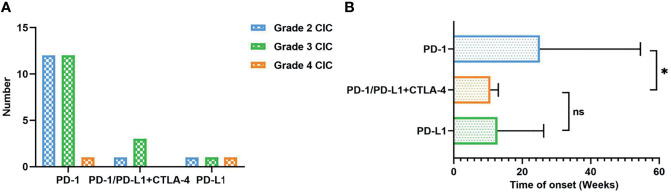
**(A)** The number of CIC patients according to different ICI therapies and severity grade. **(B)** Time of onset (weeks) classified by different ICI therapies. CIC, checkpoint inhibitor colitis; ICIs, immune checkpoint inhibitors. *P < 0.05; ns, no significant.

Nearly 20%–30% of patients would develop diarrhea after ICI therapy, while no more than 5% of patients have colitis (30). Patients treated with CTLA-4 inhibitors tend to experience three times higher CIC frequency than those with PD-1/PD-L1 inhibitors (19, 31, 32). It has been proposed that CTLA-4 blockade induces CD4^+^ T-cell activation, resulting in a generalized immune response. In contrast, PD-1/PD-L1 blockades aim at late T-cell proliferation and, therefore, cause a more localized immune reaction (33). According to previous meta-analysis and systematic reviews (30, 33, 34), the overall incidence of diarrhea was 30.2%–35.4% for CTLA-4 inhibitors and 12.1%–13.7% for PD-1/PD-L1 inhibitors. The all-grade incidence of colitis was 5.7%–39.1% for CTLA-4 inhibitors and 0.7%–31.6% for PD-1/PD-L1 inhibitors. In lung cancer, the rates of all-grade and high-grade diarrhea were 9.1%–11.0% and 0.6%–1.1% for PD-1/PD-L1 inhibitors, respectively (34). All-grade and high-grade colitis caused by PD-1/PD-L1 inhibitors separately occurred at 0.4%–0.9% and 0.1%–0.6%. The combination of PD-1/PD-L1 inhibitors and CTLA inhibitors could reach 40.4% in all-grade diarrhea/colitis. We searched PubMed and Web of Science from 2017 to October 10, 2021, and included 32 occurrences of CIC in NSCLC patients. The search term we utilized were “non-small cell lung cancer*” AND “colitis*” AND “immunotherapy*” OR “immune checkpoint inhibitors*.” All related case reports were included (**Table 1**). In our analysis, over 70% of patients were PD-1 inhibitor-related colitis, and patients treated with PD-1^+^ CTLA inhibitors were prone to suffer grade 3 CIC (**Figure 1A**).

**Table 1:** Published case reports and case series of immune checkpoint inhibitor-associated colitis.

Author	Year	Patient	Country	Cancer Type	Histologic Type	Genomic alterations (PD-1/PD-L1) (%)	Drug			Previous Therapy	CIC Grade	Time of Onset	Examination	Withdrew the Drug	Time to Withdraw the Drug	Treatment		Outcome		
							PD-1 Inhibitors	PD-L1 Inhibitors	CTLA-4 Inhibitors									CIC	CIC Course (weeks)	Other IrAEs
Kunogi et al. (35)	2021	62/M	Japan	NSCLC	AC	/	Pembrolizumab			Surgery; Chemotherapy	3	16 weeks	Colonoscopy	Yes	16 weeks	PSL; IFX; vedolizumab; TM		Deteriorated after PSL; IFX; vedolizumab; improved after TM	13	
Mourad and De Robles (36)	2021	62/M	Australia	NSCLC	Unknown	/	Pembrolizumab			Chemotherapy	2	9 weeks	CT	Yes	9 weeks	Symptomatic treatment; kept fasted	Improved (3 weeks)–recurrent (4 weeks)–deteriorated (8 weeks)		8	
Naito et al. (37)	2021	70/M	Japan	NSCLC	Unknown	/		Atezzolizumab		Chemotherapy; targeted therapy	3	5 weeks	CT/endoscopy	Yes	5 weeks	PSL	Resolved		5	Rash
Omotehara et al. (38)	2021	61/F	Japan	NSCLC	AC	/	Pembrolizumab			Surgery	3	26 weeks	CT/endoscopy	Yes	26 weeks	PSL	Improved		1	Thyroid toxicity
Hirabae et al. (39)	2020	60/M	Japan	NSCLC	AC	5%	Pembrolizumab			Chemotherapy	2	44 weeks	None	Yes	44 weeks	PSL	Resolved		4	
Grover et al. (40)	2020	54/F	USA	NSCLC	Unknown	/	Pembrolizumab			Unknown	2	7 weeks	CT	Yes	7 weeks	Steroid	Unknown		/	
Gallo et al. (41)	2020	57/F	Italy	NSCLC	AC	40%		Atezolizumab		Surgery; chemotherapy	3	2 weeks	Endoscopy	Yes	8 weeks	Probiotics; antidiarrheal drugs; antibiotics; MP	Improved (2 weeks), resolved (12 weeks), recurrence (17 weeks), maintained		52	
Yoshimura et al. (42)	2020	64/F	Japan	NSCLC	AC	100%	Pembrolizumab			Chemotherapy	3	1 week	CT; endoscopy	Yes	1 week	PSL; MP; IFX	Improved		/	
Yoshimura et al. (42)	2020	77/F	Japan	NSCLC	AC	Over 50%	Pembrolizumab			None	3	6 weeks	Endoscopy	Yes	6 weeks	Antidiarrheal drugs; PSL	Improved		0	
Yoshimura et al. (42)	2020	73/M	Japan	NSCLC	AC	Over 85%	Pembrolizumab			Unknown	2	8 weeks	Endoscopy	Yes	8 weeks	Antidiarrheal drugs; probiotics	Improved		/	
Babacan and Tanvetyanon (43)	2019	75/F	Turkey	NSCLC	NEC	/		Durvalumab	Tremelimumab	Chemotherapy	3	8 weeks	CT	Yes	8 weeks	Steroid; adalimumab; antibiotics	Improved (several days), deteriorated (2 months), resolved (a few weeks later)		12	
Babacan and Tanvetyanon (43)	2019	71/F	Turkey	NSCLC	AC	/	Nivolumab			Unknown	3	12 weeks	CT	Yes	68 weeks	Antidiarrheal drugs; antibiotics	Deteriorated (unknown)–improved (unknown)–resolved completely (after 68 weeks)		68	
Babacan and Tanvetyanon (43)	2019	72/M	Turkey	NSCLC	AC	/		Durvalumab		Target therapy	3	32 weeks	CT	Yes	32 weeks	Antibiotics; MP	Promptly improved–deteriorated (2 days)–resolved (1 week)		1 week	
Babacan and Tanvetyanon (43)	2019	69/M	Turkey	NSCLC	AC	/		Durvalumab	Tremelimumab	Chemotherapy	4	12 weeks	CT	Yes	20 weeks	Antibiotics; PS; IFX	Resolved (quickly)–recurrent (4 weeks)–resolved–recurrent (8 weeks)–resolved (16 weeks)		16	Hypophysitis
Babacan and Tanvetyanon (43)	2019	62/F	Turkey	NSCLC	AC	/	Nivolumab			Chemotherapy	2	56 weeks	CT	Yes	56 weeks	Antibiotics; PS	Resolved (4 weeks)–recurrent (taper)–improved (6 weeks)–recurrent (64 weeks)–resolved (88 weeks)		88	
Zhou et al. (44)	2019	56/F	USA	NSCLC	Unknown	/	Pembrolizumab			Chemotherapy	2	9 weeks	CT	Yes	9 weeks	Antibiotics; MP; PS	Improved (3 weeks)		3	
Deligiorgi et al. (45)	2019	72/M	Greece	NSCLC	SC	/	Nivolumab			Chemoradiotherapy	2	15 weeks	PET/CT; endoscopy	Yes	15 weeks	PSL	Resolved		6	Pneumonitis
Ni et al. (46)	2019	73/M	China	NSCLC	AC	/	Tislelizumab			Chemotherapy	3	26 weeks	CT; endoscopy	Yes	26 weeks	MP; antiviral drugs; antibiotics	Improved after MP (3 days); recurrent (4 weeks); resolved (8 weeks)		8	/
Alhatem et al. (47)	2019	78/M	USA	NSCLC	AC	/	Nivolumab			Surgery	2	48 weeks	Endoscopy	Yes	1 year	PS	Resolved		6	Gastritis
Ba´rbara; Cancela-Díez et al. (48)	2019	79/M	Spain	NSCLC	Unknown	/	Nivolumab			Chemoradiotherapy	4	21 weeks	Unknown	Yes	21 weeks	Probiotics; antidiarrheal drugs; MP	Improved (unknown time)–recurrent (8 weeks later)–deteriorated (10 weeks)		10	Coronary toxicities
Dhenin et al. (49)	2019	79/F	Belgium	NSCLC	AC	100%	Pembrolizumab			Surgery; chemotherapy	3	22 weeks	CT	Yes	22 weeks	MP	Resolved		1 day	Rash (grade 2), pericardial effusion, myasthenia gravis
Beck (50)	2019	62/F	USA	Lung cancer	AC	80	Pembrolizumab			None	3	8 weeks	CT/surgery	Yes	20 weeks	Surgery	Improved (1 week)–deteriorated (30 weeks)–improved (33 weeks)		33	/
Ibraheim et al. (51)	2019	78/F	UK	NSCLC	Unknown	/	Nivolumab		Ipilimumab	Unknown	2	12 weeks	X-ray/endoscopy	Yes	12 weeks	BM	Resolved		5	Hepatitis; pneumonitis
Yasuda et al. (52)	2018	62/M	Japan	NSCLC	AC	/	Nivolumab			Chemotherapy	3	140 weeks	CT; endoscopy	Yes	140 weeks	Antibiotics; PSL	Resolved		3	
Yamauchi et al. (53)	2018	73/M	Japan	NSCLC	Unknown	/	Nivolumab			Unknown	3	15 weeks	Endoscopy	Yes	15 weeks	Probiotics; antidiarrheal drugs; mesalazine	Improved		/	
Yamauchi et al. (53)	2018	78/M	Japan	NSCLC	AC	/	Nivolumab			Unknown	2	7 weeks	Endoscopy	Yes	7 weeks	Probiotics; antidiarrheal drugs; MP	Improved		/	
Yamauchi et al. (53)	2018	49/M	Japan	NSCLC	AC	/	Nivolumab			Unknown	2	3 weeks	Endoscopy	Yes	3 weeks	Probiotics; MP	Improved		/	
Iyoda et al. (54)	2018	62/M	Japan	NSCLC	AC	/	Nivolumab			Chemotherapy	3	38.5 weeks	Endoscopy	Yes	38.5 weeks	DM; MP; IFX; cyclosporine	Improved to grade 1 (d10); deteriorated to grade 3 (d12); did not improve (d33); improved (d63); resolved (d74), recurrent (d88)		12.6	
Callens et al. (55)	2018	63/M	Belgium	NSCLC	AC	/	Nivolumab			Chemotherapy	2	64 weeks	CT; endoscopy	Yes	64 weeks	MP; IFX	Deteriorated		9	/
Santiago; Gonzalez-Vazquez et al. (56)	2017	43/M	Spain	NSCLC	AC	/		Atezolizumab		Chemotherapy	2	12 weeks	Endoscopy	Yes	12 weeks	Unknown	Unknown		/	
Takenaka et al. (57)	2017	45/F	Japan	NSCLC	AC	/	Nivolumab			Surgery; chemotherapy; targeted therapy	2	18 weeks	CT; endoscopy	Yes	18 weeks	PSL; IFX	Resolved (3 weeks)–recurrent (24 weeks)–resolved		26	
Kubo et al. (58)	2017	82/M	Japan	NSCLC	Unknown	/	Nivolumab			Chemotherapy	3	8 weeks	Endoscopy	Yes	14 weeks	Mesalazine	Improved		4	/

CIC, checkpoint inhibitor colitis; NSCLC, non-small cell lung cancer; AC, adenocarcinoma; NEC, neuroendocrine carcinoma; SC, squamous cell carcinoma; CT, computed tomography; PSL, prednisolone; IFX, infliximab; TM, tacrolimus; MP, methylprednisolone; PS, prednisone; BM, beclometasone; DM, dexamethasone.

ICI-mediated colitis could appear at any time, such as during treatment initiation, the steroid tapering period, or after treatment termination (59). The overall onset of CIC spanned from 0 to 6.3 months (25, 53, 60–63). Prior research studies suggested that CIC onset might vary from different ICI types. Colitis induced by CTLA-4 inhibitors seemed to happen later than that caused by PD-1/PD-L1 inhibitors. The median time to the onset of ipilimumab-induced colitis was approximately 6 to 7 weeks, with an average after two to three infusions (64–68). The range of the median onset from the last injection to diarrhea was 0 to 2 months (67, 69). As for PD-1/PD-L1 inhibitors, symptomatic colitis was less predictable with the onset from nearly 1 week to 2 years (19, 70, 71). Besides, the onset of CIC seemed to be significantly earlier for patients who received combined ICIs (70). Our analysis also showed that CIC patients with PD-1/PD-L1 and CTLA-4 therapy had earlier onset than those with PD-1 inhibitors (10 vs. 25 weeks, *P* < 0.05) (**Figure 1B**). Moreover, it could also be affected by other therapies, including previous anticancer treatment, antibiotics, and non-steroidal anti-inflammatory drugs (NSAIDs) (72). The development of CIC was of great difficulty to be predicted before symptom onset (73). Lord et al. (74) suggested that the resolution time of CIC could exceed 130 days, over 10 times than the half-life period of ipilimumab. This implies the long-term influence of immunotherapy on the immune system instead of simply checkpoint blockade at the moment of treatment in the immune cascade. Therefore, we should keep continuous vigilance during the period of ICI utilization.

## Mechanism

**Figure 2:**
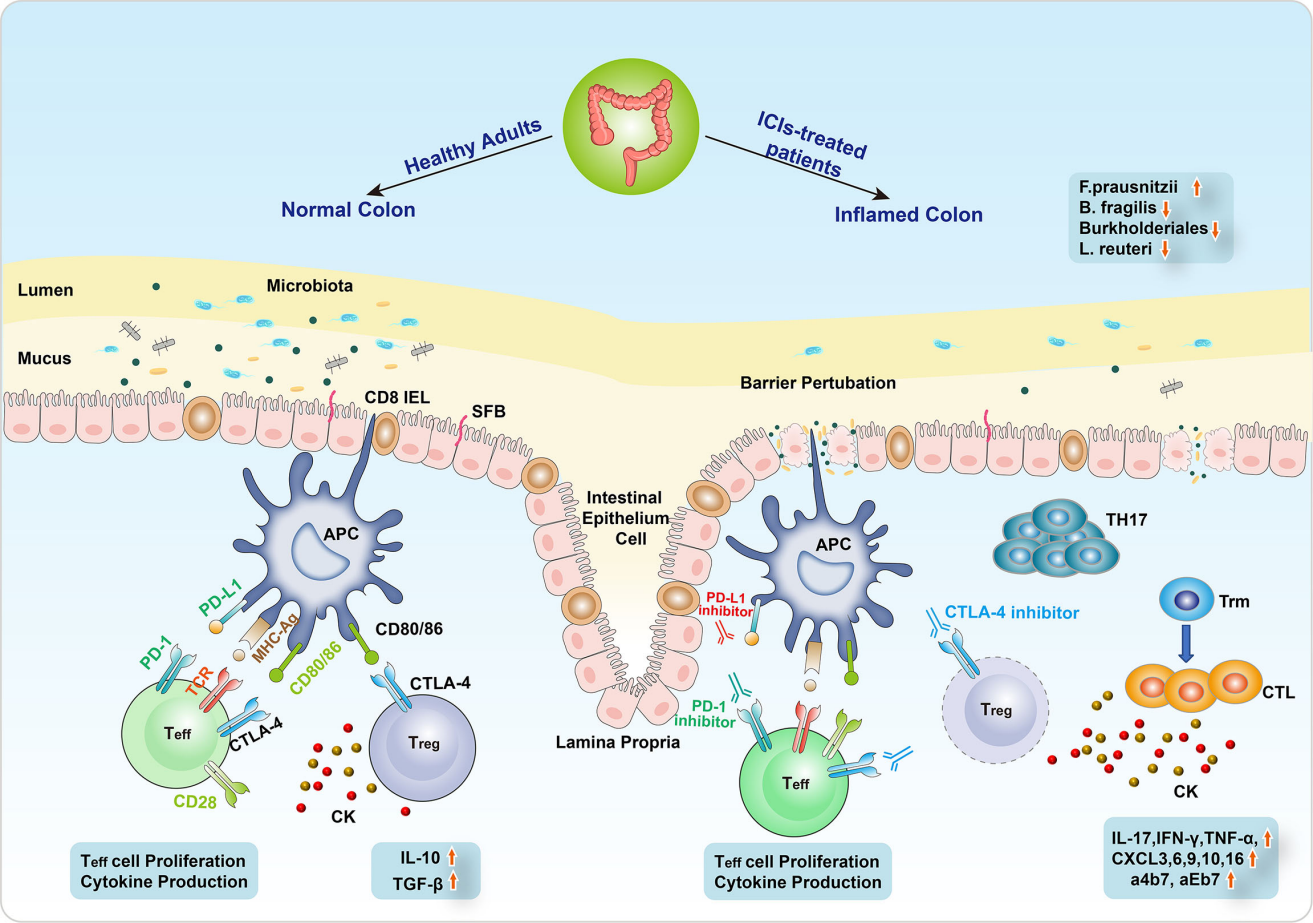
The mechanism of ICI-related colitis. The normal intestinal epithelial barrier is a highly organized mucosal surface that prevents microbiota from entering into the lamina propria. The epithelium is composed of a single layer of intestinal epithelial cells (IECs) covered by a mucus layer. Segmented filamentous bacteria (SFB) seldom breach the barrier of mucus and contact with the IECs. Immune checkpoint inhibitors (ICIs) modulate the microbiota–intestinal barrier equilibrium by inducing IEC apoptosis, resulting in barrier perturbation. The alterations of intestinal flora include the decrease of *Bacteroides fragilis*, Burkholderiales, and *Lactobacillus reuteri* and the increase of *Faecalibacterium prausnitzii*. Barrier perturbation is further aggravated possibly due to pathogenic Th17 cells, leading to severe intestinal toxicity resembling an early sign of colitis. Besides, the differentiation from CD8^+^ tissue-resident memory T (Trm) cells to cytotoxic T lymphocytes (CTLs) induced cytokines (IL-17, IFN-γ, TNF-α) to participate in the CIC emergence. In addition, CXCR3, 6, 9/10, 16, and a4b7/aEb7 receptors are also involved in the development of CIC.

The underlying mechanism of ICI-related colitis still remains obscure. However, extensive research studies have suggested that the proposed mechanisms included the hyperactivation of effector T cell (Teff cell), the infiltration of lymphocytes, and the increase of circulating memory T cells, thus causing proinflammatory status and the emergence of autoimmune-type presentation (25, 75–77). Besides, the gut microbiome also plays a critical role in the regulation of intestinal mucosal homeostasis (**Figure 2**) (78, 79).

### Mechanism of irAEs

To target and eliminate tumor cells, T cells need two stimulating signals to be activated (80–82). One is the direct antigen stimulation mediated by the T-cell receptor (TCR) and major histocompatibility complex (MHC) class II molecule on the APCs. The other depends on the co-stimulation between CD28 receptor on T cells and CD80/86 (B7) on APCs. T-cell activation results in its proliferation, cytokine production, and CTLA4 and PD1 expression (83). The role of CTLA-4, which is constitutively located on the surface of FOXP3 regulatory CD4^+^ T cells, is to terminate this co-stimulation. As a co-inhibitory ligand, CTLA-4 harbors higher affinity than CD28, contributing to competitively bind CD80/86, thus blunting the immune response. In addition to the CTLA-4-mediated inhibitory interaction between T cells and APCs, immunosuppressive cytokines produced by regulatory T cells (Treg cells) include IL-10 and TGF-β and also inhibit T-cell activation and proliferation (84, 85). Conversely, PD-1 with PD-L1 does not restrain co-stimulation but suppresses the TCR downstream signaling, which results in the reduction of transcription factors and cytokines like TBET and GATA3, as well as the enhanced inhibitory effect of Treg cells (86, 87). Both CTLA and PD-1/PD-L1 inhibitors could strengthen the activation and proliferation of Teff cells, abrogate Treg cell function, induce inflammatory cytokines, probably boost humoral autoimmunity (88), and cause a range of autoimmunity-related adverse effects in multiple organs (**Figure 2**).

### Mechanism of CIC

Several animal models with the absence of immune checkpoints have been utilized to simulate the immunological effects of CIC. Mice knocked out CTLA-4 presented diffuse immune cells infiltration in various organs and fatal colitis caused by increased T-cell activity (85, 89, 90). The PD-1 axis is of great importance in the development of innate lymphoid cells (ILCs), which have been regarded as critical effective cells at gastrointestinal mucosal surfaces (91–93). The severity of CIC was correlated to the growing mucosal number of group 3 ILCs (ILC3s) (94). Moreover, a high-level infiltration of CD4^+^ T cells and CD8^+^ T cells was reported in patients with severe colitis following immunotherapy, of which the former was more prevalent in those who underwent anti-CTLA-4 therapy and the latter usually occurred in anti-PD-1-induced colitis (95–97). Recently, a comprehensive single-cell analysis of CIC illustrated the differentiation from CD8^+^ tissue-resident memory T (Trm) cells to cytotoxic T lymphocytes (CTLs) (97). It was hypothesized that the abundant CD8^+^ Trm cells in the normal colon exerted a vital effect on CIC, and their activation prompted the subsequent assembly of CD4^+^ and CD8^+^ T cells. In addition, cytokines may participate in the emergence of CIC. The preclinical model of CIC has observed the rise of IL-17 (11, 98). The inducible gene and expression of IFN-γ and TNF-α, which induced cell death, showed a substantial increase in ICI-related colitis patients (97, 99). TNF-like cytokine 1A (TL1A) and its receptor DR3 were also upregulated in ICI-mediated colitis (100). Besides, CXCR3 and CXCR6 chemokine receptor (CXCR9/10 and CXCR16, respectively) genes presented high-level expression on colitis-related T-cell population, upregulating T-cell activity (101). Besides, three genes (*ITGB7*, *ITGA4*, and *ITGAE*), encoding a4b7 and aEb7 integrin receptors, were expressed in colitis-associated T-cell clusters. This might lead to lymphocyte retention in the intestinal mucosa (**Figure 2**) (102).

### Gut Microbiome and CIC

Immunotherapy could modulate microbiota–gut barrier equilibrium by intraepithelial lymphocyte (IEL)-mediated apoptosis of intestinal epithelial cells (IECs), leading to barrier perturbation (103). The role of dysbiosis has been proposed to initiate irAEs, in which the product exposure derived by the microbiome could induce innate immune responses, probably activating self-reactive immune cells (104). Several species of gut bacteria have been indicated to potentiate Treg cell induction, which maintained intestinal tolerance (105). CD4^+^ T helper cells induced by IL-17 (Th17), located in the lamina propria to focus specifically on intestinal-resident segmented filamentous bacteria (SFB), could deteriorate systemic autoimmune diseases under some circumstances (**Figure 2**) (106, 107).

Currently, accumulating evidence has demonstrated that gut microbiome features may be linked with the development of CIC and tumor response to ICI therapy (**Figure 2**) (77, 108–111). Chaput et al. (112) conducted a prospective study to evaluate the intestinal microbiome characteristics at baseline and the time of gastrointestinal toxicity in 26 patients who received ipilimumab. The results showed that ipilimumab could not influence the composition of the microbiome. Nonetheless, decreased microbiota diversity was found in ICI-related colitis, especially some genera like *Faecalibacterium prausnitzii* (*F. prausnitzii*) and *Bacteroides fragilis* (*B. fragilis*) in the Firmicutes phylum.

Enrichment of *F. prausnitzii* might be associated with the occurrence of colitis (112). As an obligate anaerobe, *F. prausnitzii* has the normal function of keeping colonic mucosa integrity (111). The increase of *F. prausnitzii* was linked to T-cell proliferation in the gut mucosa and facilitated the recruitment of Treg cells and a4b7 T cells. In addition, it could enhance CTL concentration in the tumor microenvironment, prolonging the OS and PFS (113). The strong and beneficial response would be presented in patients with the abundance of *F. prausnitzii* receiving anti-CTLA-4 and/or anti-PD-1 inhibitors, possibly with the cost of immunotherapy-induced colitis.

Fragilis increase is considered to be the protective factor of CIC (81, 108). It acts to play an anti-inflammatory role in the gastrointestinal tract (114). The CTLA-4 pathway was involved in the immunoregulatory process, in which *B. fragilis* produced polysaccharide A to upregulate the level of IL-10, thus alleviating inflammation (115). Moreover, the anti-CTLA-4 blockade has been verified to favor *B. fragilis* outgrowth in the colon (116). Although *B. fragilis* frequency keeps stable after anti-CTLA-4 therapy (81), the elevated number of *B. fragilis* could further decrease the size of tumor in patients treated with ipilimumab. Besides, anti-CTLA-4 antibodies could also promote dysbiosis by reducing Burkholderiales and increasing Clostridiales bacteria presentation (103, 116, 117). The combination of *Burkholderia cepacia* (*B. cepacia*) and *B. fragilis* or the administration of *Bacteroides thetaiotaomicron* (*B. thetaiotaomicron*) allowed germ-free (GF) mice to decrease the gut toxicity and elicit anticancer immune response presented in normal microbiota mice (116, 118). This might be attributed to the accumulation and maturation of plasmacytoid DCs, which produced IL-12 subsequently to stimulate ICOS^+^ Treg cells and thus promote antitumor immunity, induced by these bacteria in the gastrointestinal lamina propria. Furthermore, the response of anti-CTLA-4 inhibitors required microbiota-dependent T-cell activation. The therapeutic efficacy of the anti-CTLA-4 inhibitor could be restored by adoptively transferring *B. fragilis*-specific T cells into GF mice (117). Similar experiments conducted with PD-L1 inhibitors showed that *Bifidobacterium* species could mediate antitumor efficacy (119). This bacterium could attenuate bowel inflammation mediated by Treg cells without detriments to the antitumor response (120). Wang et al. (94) also established ICI-related colitis models to find the change of gut microbiota. The microbiota profiling showed that *Lactobacillus* presented lower abundance in the ICI-treated mice stool than in the control group. Similarly, a trinitrobenzene sulfonic acid (TNBS)-built mouse model showed that intestinal inflammation could be suppressed by specific *Lactobacillus reuteri* strains (121). In addition, *L. reuteri* could augment DC function to improve antitumor response after blocking PD-L1.

Therefore, the species and relative abundance of gut microbiota could influence the development of CIC. Some specific microbial species might orchestrate the inflammation initiation, while other subsets might help perpetuate CIC. More efforts should be dedicated to further learn the complex symbiosis between bacteria and ICI-induced colitis, with the ultimate aim to manipulate the microbiota for the balance of tumor response and irAEs.

## Risk Factors

The current risk factors for CIC cover past medical history and tumor type, therapy management, and molecular markers. Specifically, these included medication history, preexisting autoimmune diseases, tumor type, combined ICI therapy, and inflammatory cell levels.

### Past Medical History and Tumor Type

The utilization of NSAIDs has been regarded to increase the risk of CIC (77). Patients with CIC (31%) were more common to use NSAIDs than those without CIC (5%) (*P* = 0.003), which is similar with IBD, and there is a correlation between NSAIDs and increased IBD flare risk (122). Conversely, vitamin D use was found to reduce the risk for ICI-related colitis (123, 124). In addition, the risk of CIC of patients who had previous irAEs is controversial. Some studies suggested that patients who had irAEs with the first ICI initiation would experience a higher risk of irAEs following alternate ICI therapy (125, 126). However, other studies showed that patients did not develop recurrent severe diarrhea or CIC when they changed to subsequent PD-1/PD-L1 inhibitor therapy after ipilimumab (127, 128). Moreover, the occurrence of CIC might relate to malignancy types. Melanoma seemed to have a higher risk of CIC compared with renal carcinoma and NSCLC (33). However, the reason behind the discrepancy between the rates from these two cancer types remains obscure.

Autoimmune disorders, including IBD, were not always considered in ICI clinical trial criterion, since the disease might worsen by non-specific T-cell activation of ICIs. Nearly 27% of patients have underlying disease exacerbation after ICI therapy, which means increased or recurrent prior manifestations (129). Few studies demonstrated that patients with preexisting autoimmune diseases would experience a higher incidence of CIC (129, 130). The occurrence of CIC in underlying IBD patients who received ipilimumab was reported at 30%. Besides, Menzies et al. (126) reported that five cases with prior IBD history who received PD-1 blockade had great tolerance without disease flare. Therefore, PD-1 inhibitors are more recommended for IBD patients than ipilimumab. Currently, a prospective clinical protocol is recruiting IBD patients who have ICI indications (AIM-NIVO, NCT03816345). This would bring critical instruction for ICI therapy of IBD patients in evaluating risk and developing decision-making.

### Therapy Management

The combination of ICI therapy is reported to present a higher occurrence of ICI-related colitis. A recent meta-analysis included 27 prospective studies reporting the risk of CIC in patients with lung cancer who received ICI therapy (31, 131). The results showed that the combination of anti-CTLA-4 and anti-PD-1 therapy had a higher risk of all grades of CIC than anti-PD-1 monotherapy (RR: 1.61, 95% CI: 1.14–2.29). The combined ICIs, such as ipilimumab and pembrolizumab/nivolumab (132) and durvalumab and tremelimumab (133, 134), obtained above 10% occurrence of colitis (79, 135). Moreover, the sequential administration of ipilimumab before nivolumab showed higher rates of grade 3 colitis than nivolumab before ipilimumab (16% vs. 10%) (136). No particular toxicities were found in the combination therapy (137). The same class of ICI therapy had a similar incidence of all grades of CIC. However, the combination of chemotherapy/radiotherapy and immunotherapy could not increase the incidence of colitis but could promote tumor response rates, PFS, and OS in NSCLC (138, 139).

### Molecular Markers

Several potential molecular markers, such as increased IL-17, neutrophil, eosinophil, and WBC levels, have merged to predict the development of ICI-mediated colitis (31, 140–142). Decreased baseline granulocyte colony-stimulating factor (GCSF) level was found in CIC patients (143, 144). Moreover, the association between some cell surface markers and GI-irAEs was also investigated. Shahabi et al. (145) explored the gene expression profiling of whole blood in 162 patients who were treated with ipilimumab. They found that pretreatment elevated the baseline levels of genes involved in cell cycle (*WDR1*, *FP36L2*, *PCGF1*, *BAT1*, *BANF1*, and *SPTAN1*), vesicle trafficking-correlated genes (*VAMP3*, *SNAP23*, and *PICALM*), and immune-related genes (*RAC2*, *IL-32*, *IL2RG*, *CD37*, *CD4*, and *CD3E*), and these were associated with developing GI-irAEs. After 3 weeks of treatment, the biomarker which distinguished GI-irAEs and non-GI-irAEs was CD177, a specific neutrophil surface biomarker that exerts effects in activating neutrophils and recruiting inflammatory cells. However, it could not be utilized alone to predict irAE occurrence because of large individual variability and its low sensitivity. Besides, CEA-associated cell adhesion molecule (CEACAM), as the essential adherent mediator in the migration of neutrophils, also showed a significant increase in the ICI-related GI toxicity group. Therefore, the changes in CEACAM and CD177 expression might become more sensitive markers than the absolute neutrophil level in the peripheral blood, and these activation steps happen in the early neutrophil recruitment.

## Manifestations

### Clinical Manifestations

The majority of clinical trials discriminate diarrhea from colitis, although they overlap in the most practical cases. Diarrhea is evaluated by stool increase per day. Colitis is evaluated according to clinical symptoms (blood or mucus in stool, abdominal pain) or the diagnostic findings on the imaging and endoscopic observations (146). The grading of diarrhea/colitis was according to the Common Terminology Criteria for Adverse Events v5.0 (147).

**Figure 3:**
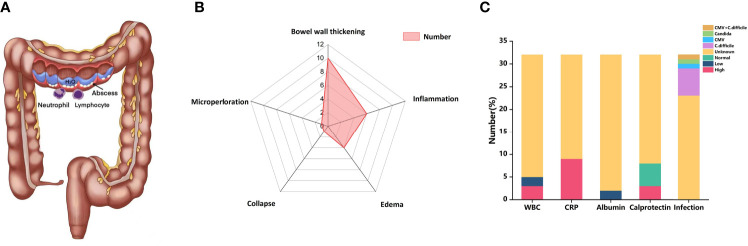
**(A)** CIC physically presented more similarly with colitis than IBD, involving lymphocyte and neutrophil infiltration. **(B)** Radar chart of CT features of CIC. **(C)** Bar chart of laboratory features of CIC. CIC, checkpoint inhibitor colitis; ICIs, immune checkpoint inhibitors; WBC, while blood cells; CRP, C-reactive protein; CMV, cytomegalovirus; *C. difficile, Clostridium difficile*.

Similar to IBD, diarrhea is the most common clinical symptom of CIC (**Figure 3A**). It is also characterized by abdominal pain, hematochezia, fever, vomiting, nausea, and loss of appetite (77). Intestinal perforation and weight loss are less common (148). In our analysis, almost all patients developed diarrhea, ranging from 4 to 30 times. Additionally, other common clinical manifestations also include abdominal pain (25%), loss of appetite (18.75%), and hematochezia (12.5%). Obstipation, tachycardia, and malnutrition were also reported in our patients with CIC.

### Imaging Manifestations

CT and/or MRI is the major radiology examination for CIC. However, CT presents a relatively low negative predictive value, with 53%–85% sensitivity and 75%–78% specificity (22, 149, 150). Colonic mucosal hyperenhancement is the most common CT sign of CIC, followed by mesenteric vessel engorgement, bowel wall thickening, fluid-filled distended colon, pericolic fat stranding, and bowel wall edema (151). In our analysis, the most common features of CT in our analysis were bowel wall thickening (10/32) (**Figure 3B**). Universally, the patterns of CIC could be divided into pancolitis, segmental colitis associated with diverticulosis (SCAD) enterocolitis, and enteritis (152–154). It is reported that the different CT patterns of CIC are related to corresponding clinical features. For example, patients with diffuse colitis usually have watery diarrhea, while patients with SCAD pattern have mixed watery and bloody diarrhea and cramping pain (154). When severe colitis was complicated with bowel perforation and toxic megacolon, CT would be an effective tool for diagnosis (155). The sign of non-dependent extraluminal air close to the intestine might be conducive to bowel perforation. PET-CT was also applied in CIC patients with increased 2-deoxy-2-(18F) fluoro-D-glucose (FDG) uptake (50, 150). One of our cases showed colon inflammatory activity when PET/CT was performed (45).

### Laboratory Features

The serological findings of patients with CIC are not specific. Some literature has reported that markers such as C-reactive protein (CRP) or low serum albumin level counts increased in the serum of IMC patients, but this may be influenced by systemic inflammation. In a retrospective study by Abolhassani et al., 88 patients with advanced melanoma were diagnosed irAEs after receiving ICIs, and 42% of them had a high CRP level (156). In our analysis, 9 of 32 patients had an increased level of CRP, and two patients showed low albumin levels (**Figure 3C**). Other serum findings may also be related to CIC, such as increased IL-17 (157), eosinophil count (158), and neutrophil–lymphocyte ratio. Some patients with CIC may show anemia on serological findings because of chronic bleeding in the digestive tract (77). In our analysis, three patients showed a decreased level of WBC (**Figure 3C**). Besides, elevations were found in serum leucine-rich α2-glycoprotein (LRG) and creatinine (42). LRG was reported to distinguish different colitis forms like drug-related and *Cytomegalovirus* (CMV) colitis (42).

Fecal calprotectin is often elevated in the stool specimens of patients developing IMC, which is a predictor of disease activity and prognosis. We found two patients with CIC had elevated fecal calprotectin (**Figure 3C**). Zou et al. (159) retrospectively analyzed the fecal calprotectin of patients with CIC and found that it was increased at the onset of IMC while decreased after treatment. In terms of infectious pathogens, *Clostridium difficile* (*C. difficile*) was the most common pathogen. CMV and *Candida* were also reported in our included patients (**Figure 3C**).

### Endoscopic Features

**Figure 4:**
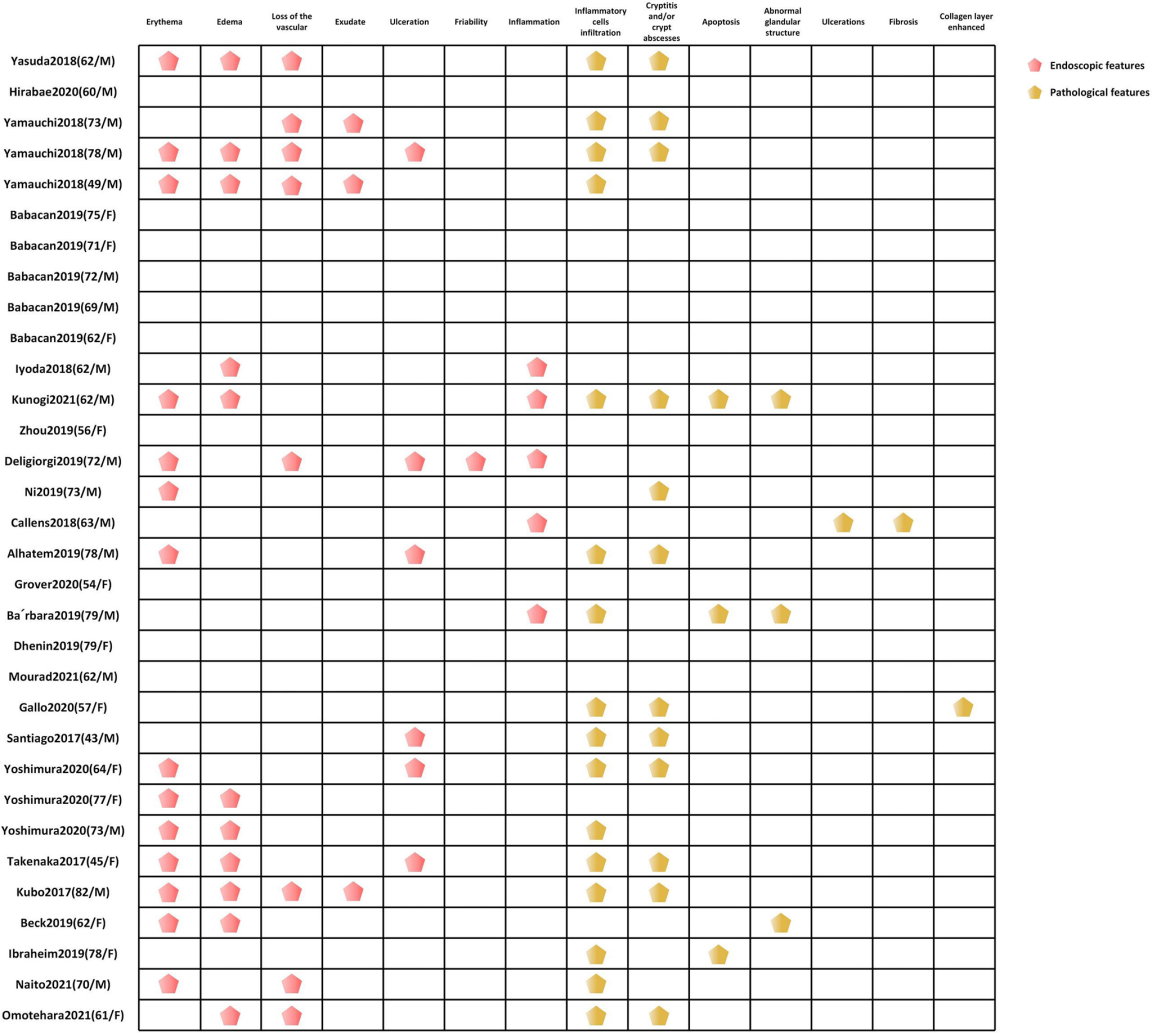
The endoscopic and pathological features of CIC. CIC, checkpoint inhibitor colitis.

Early endoscopy examination is encouraged for diagnosis and prognostic assessment if CIC is suspected (22). Left-sided colitis (31%–43%) is the most commonly reported in CIC cases, followed with pancolitis (involvement of ≥3 segments) (23%–40%) and ileitis (11%–14%) (160, 161). Endoscopic inflammatory findings usually present with mucosal ulceration and non-ulcerative inflammation with similar incidence. Non-ulcerative inflammation is characterized by erythema, exudate, erosion, friability, loss of vascular pattern, and edematous or granular mucosa (58, 72, 79). Among these, erythema (14/32) and edema (11/32) were the most common endoscopic features in our CIC patients (**Figure 4**). Most inflammation changes are predominantly diffuse instead of patchy (73). However, half of the diarrhea patients were observed to have patchy distribution endoscopically (77), which resembles the presentation of IBD (162), especially ulcerative colitis (UC) (53, 58). Fewer individuals with CIC (35%) were found to have segmental performance, as what often appeared in Crohn’s disease (77). There is no particular grading standard for the endoscopic manifestations in CIC. Nonetheless, the Mayo score, the van der Heide score for UC, or Simple Endoscopic Score (SES) for Crohn’s disease could be utilized (61, 163, 164).

Several studies illustrated that clinical symptoms were not significantly associated with endoscopic manifestations (22, 165). We also conducted a correlation analysis between CIC grade and endoscopic (*P* = 0.581)/histological features (*P* = 0.821), with no significant difference found (**Table 2**). However, the endoscopic findings, such as ulceration and pancolitis, are the indicative factors for steroid-refractory colitis (61). The findings in the endoscope could be categorized as low risk and high risk for steroid response. High-risk presentations were defined as either ulceration larger than 1 cm and/or deeper than 2 mm or extensive colitis from the proximity to the splenic flexure of the colon (166). High-risk patients need frequent utilization of biologics (vedolizumab or infliximab). Besides, they were significantly correlated with longer hospital stays, more disease recurrence, and more requirements for the repeat endoscopy (167). Furthermore, CIC patients who received colonoscopy within 1 week of onset would experience a significantly shorter period of symptoms and steroid therapy (166).

**Table 2:** Correlation of the grade of CIC with endoscopic features and histological features.

	Total (*N* = 32)	Grade 2 (*N* = 14)	Grades 3–4 (*N* = 18)	*P*-value
**Endoscopic features**				0.581
None	12 (37.50%)	6 (42.86%)	6 (33.33%)	
Inflammation	20 (62.50%)	8 (57.14%)	12 (66.67%)	
**Histological features**				0.821
None	13 (40.60%)	6 (42.86%)	7 (38.89%)	
Inflammation	19 (59.40%)	8 (57.14%)	11 (61.11%)	

Data were collected from studies in **Table 1**.

CIC, checkpoint inhibitor colitis.

### Pathological Features

The pathological features of CIC present from focal active colon injury with patchy crypt abscess to diffuse mucous inflammation (95). We found that inflammatory cell infiltration (50%), cryptitis, and/or crypt abscesses (33.3%) were the most frequently reported pathological features (**Figure 4**). These presentations might precede the onset of clinical symptoms. Coutzac et al. (95) included asymptomatic patients who received colonoscopy 1 to 2 weeks after treatment with ipilimumab and found that colon inflammation existed before the symptom development, although most of the symptoms of the patients occurred 3 weeks later. When they ultimately presented with symptomatic diarrhea or colitis, the histological observations exhibited more severe lamina propria infiltration. CIC pathological patterns could be summarized as follows: active colitis, chronic active colitis, microscopic (collagenous and lymphocytic) colitis, ischemic colitis, increased apoptosis, and non-specific inflammatory reactive changes (168–170). Patterns overlapping might exist in the microscopic presentation of CIC.

Active colitis is the most frequent pattern of histopathologic finding in ICI-mediated colitis. The typical manifestations of active colitis are lamina propria neutrophilic infiltrate, crypt abscesses, and neutrophilic cryptitis, with or without crypt dropout/atrophy (171, 172). The atrophic crypt epithelium is attenuated, which admixes apoptotic debris and inflammatory cell (173), resembling mycophenolate-caused colitis. Granulomas accompanied with crypt rupture have also been found (32, 174). Intraepithelial lymphocytes and increased apoptosis might or might not appear.

Microscopic colitis means lymphocytic and collagenous colitis (95), which has been found in 12% of CIC cases (66, 79, 175). Usually, lymphocytic colitis has the histological characteristics of a normal crypt structure, with increased lymphoplasmacytic inflammation in the lamina propria and intraepithelial lymphocyte infiltration, but no significant acute inflammatory presentation (176, 177). Collagenous colitis is an infrequent manifestation of CIC, which has increased intraepithelial lymphocytes and thickened subepithelial collagen (66). However, the atypical feature of microscopic colitis has also been reported, like crypt abscesses and neutrophilic cryptitis of active IBD (178). Gallo et al. (41) reported a patient with lung cancer who received atezolizumab presented collagenous colitis along with acute neutrophilic inflammation. Besides, Choi et al. (179) showed that patients with microscopic colitis induced by ICIs would have higher hospitalization rates and more aggressive course of disease and need more treatments like steroids and/or immunosuppressants than those without exposure to ICIs.

Chronic active colitis, one of the pathological patterns of IBD, has also been reported in ICI-mediated colon inflammation, particularly in patients with recurrent ICI therapy. This type is characterized by increased basal plasmacytosis and lymphoplasmacytic infiltration in the lamina propria, along with acute inflammation presentation of crypt abscess/cryptitis (172). The chronic manifestations, Paneth cell or pseudo-pyloric metaplasia and crypt structural distortion, constantly appear (63). The chronic feature could appear simultaneously with active colitis or develop gradually based on it. Yamauchi et al. (53) reported that three cases with colitis induced by nivolumab exhibited similar endoscopic and histopathologic features with ulcerative colitis at the initiation of diagnosis, and therapy for ulcerative colitis was applied successfully on these patients. Besides, one study (172) found that nearly half of patients who appeared with active colitis initially developed chronic features mentioned above. Therefore, ICI-related colitis has been considered as a specific IBD form (77). Approximately 60% of patients could have chronic inflammatory presence in the pathological section of ICI-induced colonic injury (22, 32, 180).

Increased crypt epithelial apoptosis always occurs in concurrence with other CIC patterns, including lymphocytic colitis, active colitis, and chronic active colitis. Isolated increased apoptosis with the absence of active and/or chronic inflammatory manifestation, imitating graft-versus-host disease, is less common (169). Ischemic colitis is also a rare pattern of CIC, presenting with atrophic crypts, reactive epithelial changes, and fibrosis in the lamina propria (32). In addition, non-specific inflammatory reactive changes, including lymphoplasmacytic infiltrate increase in the lamina propria along with epithelial attenuation and reactive changes and depleted mucin, are also presented in the colon injury pattern.

## Diagnosis

New emerging abdominal symptoms after immunotherapy for NSCLC, especially acute diarrhea, need consideration of CIC (181). However, few patients only present with abdominal pain instead of diarrhea (50). Typically, the diagnosis of CIC is made by exclusion of infection, IBD, and tumor metastasis. Fecal etiology helps to rule out intestinal microbial infection. Endoscopy with biopsy, contributing to evaluate inflammatory severity, has been considered as the gold standard criteria for the diagnosis of ICI-related colitis. In addition, imaging examination like CT also assists in diagnosing serious complications such as toxic megacolon and intestinal perforation.

The most common differential diagnoses include infectious intestinal diseases and IBD. The infectious pathogens, such as CMV, *Salmonella*, *C. difficile*, and parasites, should be examined foremost for the diagnosis of CIC. It is worthy to notice that CIC could develop with bowel infection at the same time. One of our cases developed CIC that coexisted with CMV and *Salmonella* (46). Therefore, in addition to etiology detection, endoscopic biopsy is essential for accurate diagnosis. Moreover, tumor immunity could be triggered by blocking the pathways of PD-1/PD-L1/CTLA-4, while the immunity to infection could be inhibited. Therefore, several cases were infected with *C. difficile* after CIC (43). Under this condition, CIC would get worse, and it is necessary to add antibiotics in time. Besides, the pathogenesis and endoscopic manifestations of CIC resemble those of IBD (162, 166). However, there still exist differences in pathological appearance between CIC and IBD. Histologically, CIC is characterized with active colitis, such as neutrophilic infiltration, crypt microabscesses, and prominent crypt epithelial cell apoptosis, while IBD usually shows chronic inflammation signs, including basal lymphocytosis, crypt architectural irregularity, and Paneth cell metaplasia (173). In addition, compared with UC, CIC showed less basal plasmacytosis (14% vs. 92%), less crypt distortion (23% vs. 75%), and more apoptotic bodies ((17.6 ± 15.3 vs. 8.2 ± 4.2) (182). It is noteworthy that the clinical presentations could distinguish these different clinical entities when the histologic findings overlap, including the onset of symptoms soon after ICI initiation and other concurrent irAEs (25). Moreover, recent research illustrated distinct immunological features of colonoscopy from CIC and IBD patients, with predominantly CD4^+^ T cells and Treg cells in the anti-CTLA-4-induced colitis and IBD patients, respectively (95).

In summary, the precise diagnosis of CIC requires multidisciplinary discussion and persistent monitoring, which also plays a critical role in grade assessment.

## Management

At present, minimal prospective trials have been designed to evaluate the optimal therapeutic modality for CIC. Consequently, current management guidelines are on the strength of expert opinion and retrospective studies, recommending systemic corticosteroids as the first-line therapy uniformly (19, 71, 183). The treatment is ideally initiated not exceeding 5 days of symptom appearance (23). The specific management is mainly based on severity degree. Pretreatment examinations include infectious etiology (stool parasitism and calprotectin), blood testing (C-reactive protein, celiac disease serology, comprehensive metabolic panel, complete blood count, etc.), hepatitis serology, and tuberculosis testing, which are beneficial for monitoring treatment response and preparing for biologics if corticosteroids do not work (184).

### Steroid

Systemic high-dose glucocorticoids are typically effective for CIC cases, equaled to prednisone of 1–2 mg/kg/day, obtaining nearly 90% efficacy (185).

#### Steroid Therapy Classified by Grade

For grade 1 diarrhea/colitis, supportive treatments, such as anti-motility agents (atropine/diphenoxylate, loperamide), adequate hydration, and dietary adjustments (low-fiber, bland diet), are recommended (186). Budesonide has been proposed to reduce symptoms in patients with ICI-mediated colitis (19). ICIs could continue to be utilized without delay or interruption in the management of colitis.

For grade 2 diarrhea/colitis, ruling out the infectious factors, systemic corticosteroids (prednisone/intravenous methyl-prednisolone of 1–2 mg/kg/day) should be initiated until symptom improves to grade 1 or less. After that, the taper of steroids is recommended for at least 4 to 6 weeks (186). Endoscopy and abdominal CT should be considered strongly. In terms of ICIs, anti-CTLA-4 agents should be discontinued permanently, while anti-PD-L1 agents could be continuously held. Besides, PD-L1 inhibitors also could be resumed as monotherapy when symptoms have improved/resolved, or prednisone is reduced to 10 mg/day or less (19, 183).

For grade 3 to 4 diarrhea/colitis, the discontinuation of ICIs should be immediate and permanent (19, 71, 183, 187). Patients are usually admitted to the hospital for urgent evaluation and continuous monitoring. Intravenous methylprednisolone of 1–2 mg/kg is the prompted initial therapy (24, 79). It is also necessary to keep electrolyte balance and implement aggressive fluid resuscitation for grade 3–4 CIC patients. The corticosteroid response for grade 2–4 toxicity should be assessed early after 2–3 days. Non-responders are recommended for escalation to a biological agent.

**Figure 5:**
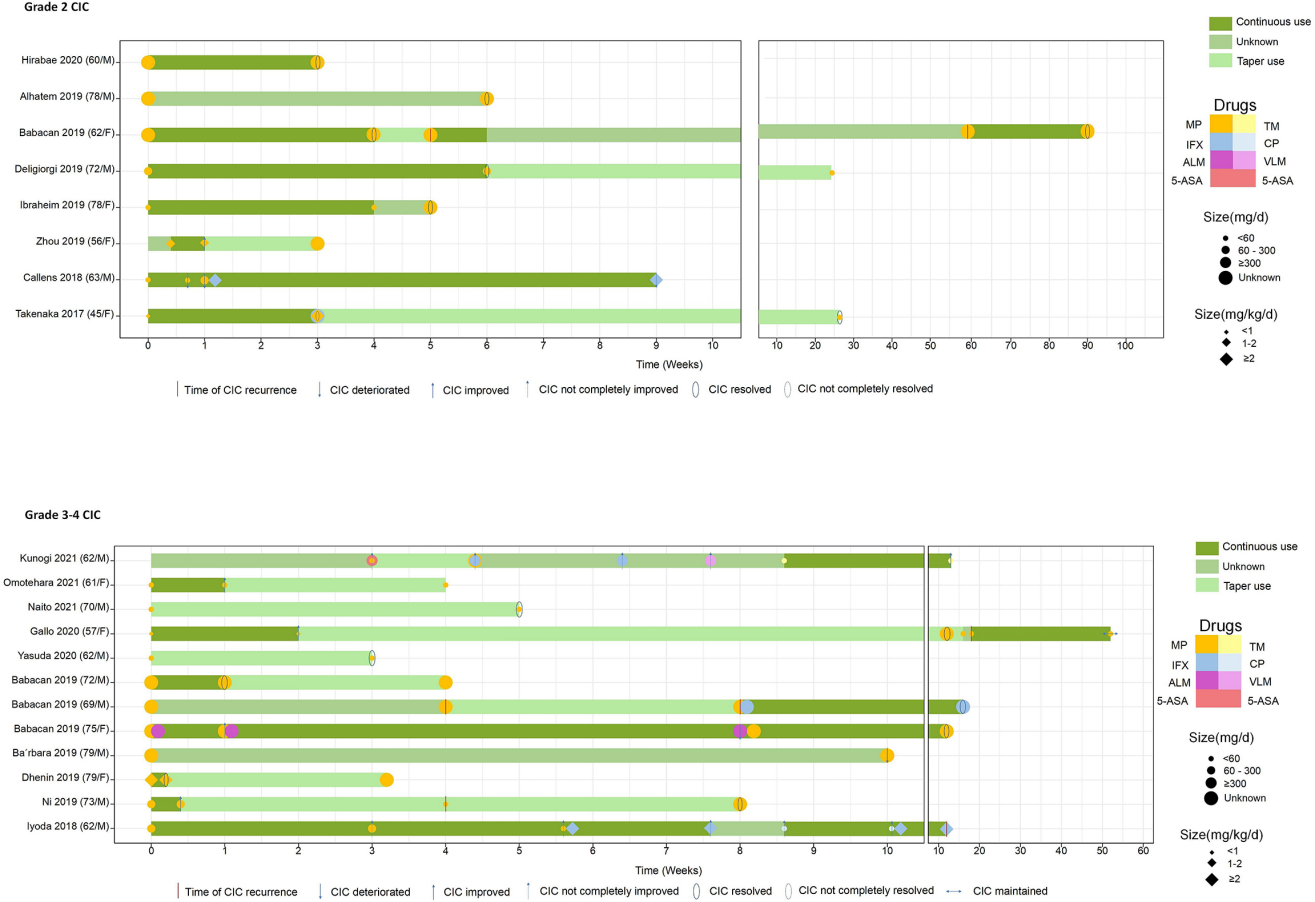
The drugs that every case utilized and the definite continuous and taper time. All the steroid doses were converted to equivalent MP. MP, methylprednisolone; TM, tacrolimus; IFX, infliximab; CP, cyclosporine; ALM, adalimumab; VLM, vedolizumab; 5-ASA, 5-aminosalicylic acid.

We also investigated the therapeutic features stratified by CIC grade (**Table 3**) and depicted the steroid duration and taper (**Figure 5**). We unified different steroid types to methylprednisolone (MP) equivalents and further classified them into high-dose, intermediate-dose, and low-dose groups, based on the initial administered dose. Since we observed that not all cases pointed out the weight of the patients, two distinct steroid dose specifications (mg/kg/day and mg/day) were established to describe the utilization of steroid. Our data showed that the median steroid initial dose was nearly 60 mg/day or 1 mg/kg/day (MP), with no significant difference found in grade 2 and 3–4 CIC patients (**Table 3**). In terms of dose group, low-dose steroid was more frequently used in grade 3–4 CIC patients, while high-dose steroid was more common in grade 2 CIC patients, although no significant discrepancy existed (**Table 3**). Extensive sample size studies with more specific steroid data are required to illustrate steroid use.

**Table 3:** The characteristics related to the management of CIC stratified by grade of CIC.

Grade of CIC Mean ± SD/*N* (%)	Total	Grade 2	Grades 3–4	*P*-value
*N*	32	14	18	
Steroid initial dose (mg/day)	63.00 ± 37.45	66.20 ± 36.17	61.00 ± 40.55	0.819
Steroid initial dose groups (mg/day)				0.071
Low dose <60	9 (69.23%)	2 (40.00%)	7 (87.50%)	
60 ≤ intermediate dose <300	4 (30.77%)	3 (60.00%)	1 (12.50%)	
Steroid initial dose (mg/kg/day)	1.37 ± 0.58	1.57 ± 0.57	1.23 ± 0.60	0.394
Steroid initial dose groups (mg/kg/day)				0.287
Low dose <1	5 (50.00%)	1 (25.00%)	4 (66.67%)	
1 ≤ intermediate dose <2	1 (10.00%)	1 (25.00%)	0 (0.00%)	
High dose ≥2	4 (40.00%)	2 (50.00%)	2 (33.33%)	
Steroid taper time (weeks)	5.64 ± 5.77	10.50 ± 9.47	3.70 ± 1.89	**0.041**
Antibiotics				**0.025**
No	23 (71.88%)	14 (87.50%)	7 (50.00%)	
Yes	9 (28.12%)	2 (12.50%)	7 (50.00%)	
Biological agents				0.36
No	25 (78.12%)	12 (85.71%)	13 (72.22%)	
Yes	7 (21.88%)	2 (14.29%)	5 (27.78%)	
OS				0.244
Alive	27 (84.38%)	13 (92.86%)	14 (77.78%)	
Dead	5 (15.62%)	1 (7.14%)	4 (22.22%)	
Survival weeks	75.26 ± 90.53	60.30 ± 71.06	86.77 ± 104.44	0.5
CIC course (weeks)	15.74 ± 22.22	17.11 ± 27.49	14.97 ± 19.62	0.823
CIC outcome				0.232
Resolved/improved	16 (53.33%)	8 (66.67%)	8 (44.44%)	
Deteriorated/recurrent	14 (46.67%)	4 (33.33%)	10 (55.56%)	
Tumor response				0.461
Complete response	1 (3.12%)	1 (6.25%)	0 (0.00%)	
Partial response	8 (25.00%)	3 (18.75%)	5 (35.71%)	
Tumor progressed	3 (9.38%)	2 (12.50%)	1 (7.14%)	
Stable	18 (56.25%)	10 (62.50%)	8 (57.14%)	
Recurrence times				0.834
0	24 (75.00%)	11 (78.57%)	13 (72.22%)	
1	6 (18.75%)	2 (14.29%)	4 (22.22%)	
2	2 (6.25%)	1 (7.14%)	1 (5.56%)	
Rechallenge times				0.49
0	28 (87.50%)	12 (85.71%)	16 (88.89%)	
1	3 (9.38%)	2 (14.29%)	1 (5.56%)	
2	1 (3.12%)	0 (0.00%)	1 (5.56%)	

Data were collected from studies in **Table 1**.

CIC, checkpoint inhibitor colitis; OS, overall survival. Bold values: P < 0.05 which means significant differences between groups.

#### The Quantifying, Taper, and Maintenance of Steroid

Limited data underscore the quantifying of steroids in treating ICI-related diarrhea/colitis. A retrospective study (188) included 90 patients with ICI-induced gastroenterocolitis and divided them into three groups based on steroid dose: “limited or none,” prednisone equivalents <7.5 mg/day on average for over 2 months; “moderate,” prednisone equivalents >7.5 mg/day on average for over 2 months; and “high,” prednisone equivalents ≥1 mg/kg/day for over 1 week (189, 190). They found that the high-dose steroid group had decreased PFS (HR = 2.54, 95% CI: 1.11–5.80, *P* = 0.03), while OS was not influenced by systemic steroid treatment for gastroenterocolitis mediated by ICIs, after the adjustment of covariates. Another study focused on the maintenance of steroid management (191). The results showed that grade 3 CIC has a median average steroid duration of 58 days. Overall grade steroid duration was 94 days, twice than grade 3 or 4 CIC steroid duration. This placed quite a few patients at risk of iatrogenic complications such as mood change and infection. Therefore, continuous supportive care during the utilization of steroid should be reinforced as well (192). The recommended taper time was 4 to 8 weeks (193, 194). The cases we included had a median steroid taper duration at nearly 5 weeks, with grade 2 and grade 3–4 CIC at about 10 and 5 weeks, respectively (*P* = 0.041) (**Table 3**). In 20 CIC patients with exact steroid data, 9 of them chose the steroid decrement after the improvement or resolution of CIC, and the duration of continuous steroid use varied from 3 days to 12 weeks (**Figure 5**).

### Biological Agents

Approximately 30%–60% of ICI-reduced diarrhea/colitis patients appear recalcitrant to first-line glucocorticoid, showing no response to high-dose steroid within 72 h of onset or no complete response in 1 week (61). They should be considered for second-line immunosuppressants. Besides, patients who suffer relapses during the steroid decrement or after finishing the steroid course also require additional immunosuppression (28), with the recurrent incidence of 44% for CTLA-4 inhibitors and 34% for PD-1/PD-L1 inhibitors (195). Similarly, our analysis also suggested that patients were more likely to receive biological agents (42.86% vs. 6.25%, *P* = 0.018) if they suffered from a deteriorated or recurrent CIC outcome rather than an improved or resolved outcome (**Table 4**). Additionally, colon ulceration, pancolitis, and high van der Heide or Mayo score are identified as the predictive factors correlated to the utilization for secondary immunosuppression (22, 61, 167). In addition, the biopsy specimens of CIC patients who required infliximab showed higher CD68 scores and CD8/FoxP3 ratios than those who responded to steroid (196).

**Table 4:** Baseline characteristics of the NSCLC cases with CIC we included.

CIC outcome Mean ± SD/*N* (%)	Total	Improved/Resolved	Deteriorated/Recurrent	*P*-value
*N*	30	16	14	
Age	66.34 ± 10.23	69.50 ± 9.67	65.29 ± 8.65	0.222
Sex	30	16	14	0.765
Female	12 (40%)	6 (50%)	6 (50%)	
Male	18 (60%)	10 (55.56%)	8 (44.44%)	
Genomic alterations (%)	65.71 ± 35.41	68.00 ± 40.71	60.00 ± 28.28	0.434
Grade of CIC				0.20
Grade 2	14 (43.75%)	8 (50.00%)	4 (28.57%)	
Grade 3	16 (50.00%)	8 (50.00%)	8 (57.14%)	
Grade 4	2 (6.25%)	0 (0.00%)	2 (14.29%)	
Histologic type				0.36
AC	22 (68.75%)	10 (62.50%)	11 (78.57%)	
NEC	1 (3.12%)	0 (0.00%)	1 (7.14%)	
SC	1 (3.12%)	1 (6.25%)	0 (0.00%)	
Unknown	8 (25.00%)	5 (31.25%)	2 (14.29%)	
Dose of onset	10.47 ± 14.01	11.75 ± 17.83	10.07 ± 9.51	0.452
Time of onset	22.20 ± 26.81	23.06 ± 34.07	23.04 ± 18.62	0.261
Steroid initial dose (mg/day)	63.00 ± 37.45	57.22 ± 27.52	76.00 ± 57.13	0.428
Steroid initial dose groups (mg/day)				0.764
Low dose <60	9 (69.23%)	6 (66.67%)	3 (75.00%)	
60 ≤ intermediate dose <300	4 (30.77%)	3 (33.33%)	1 (25.00%)	
Steroid initial dose (mg/kg/day)	1.37 ± 0.58	1.41 ± 0.60	1.27 ± 0.64	0.736
Steroid initial dose groups (mg/kg/day)				0.7
Low dose <1	5 (50.00%)	3 (42.86%)	2 (66.67%)	
1 ≤ intermediate dose <2	1 (10.00%)	1 (14.29%)	0 (0.00%)	
High dose ≥2	4 (40.00%)	3 (42.86%)	1 (33.33%)	
Steroid taper time	5.64 ± 5.77	5.50 ± 5.21	5.75 ± 6.52	0.896
Antibiotics				**0.025**
No	23 (71.88%)	14 (87.50%)	7 (50.00%)	
Yes	9 (28.12%)	2 (12.50%)	7 (50.00%)	
Biological agents				**0.018**
No	25 (78.12%)	15 (93.75%)	8 (57.14%)	
Yes	7 (21.88%)	1 (6.25%)	6 (42.86%)	
OS				0.513
Alive	25 (83.33%)	14 (87.50%)	11 (78.57%)	
Dead	5 (16.67%)	2 (12.50%)	3 (21.43%)	
Survival weeks	75.26 ± 90.53	52.92 ± 89.69	114.56 ± 86.16	**0.015**
CIC course	15.74 ± 22.22	3.36 ± 2.06	25.47 ± 26.01	**<0.001**
Tumor response				0.587
Complete response	1 (3.12%)	1 (6.25%)	0 (0.00%)	
Partial response	8 (25.00%)	3 (18.75%)	5 (35.71%)	
Tumor progressed	3 (9.38%)	2 (12.50%)	1 (7.14%)	
Stable	18 (56.25%)	10 (62.50%)	8 (57.14%)	
Recurrence times				**<0.001**
0	24 (75.00%)	16 (100.00%)	6 (42.86%)	
1	6 (18.75%)	0 (0.00%)	6 (42.86%)	
2	2 (6.25%)	0 (0.00%)	2 (14.29%)	
Rechallenge times				**0.037**
0	26 (86.67%)	16 (100.00%)	10 (71.43%)	
1	3 (10.00%)	0 (0.00%)	3 (21.43%)	
2	1 (3.33%)	0 (0.00%)	1 (7.14%)	

Data were collected from studies in **Table 1**.

NSCLC, non-small cell lung cancer; CIC, checkpoint inhibitor colitis; AC, adenocarcinoma; NEC, neuroendocrine carcinoma; SC, squamous cell carcinoma. Bold values: P < 0.05 which means significant differences between groups.

Current studies demonstrate that infliximab (IFX) and vedolizumab are the primary therapeutic approaches for steroid-refractory CIC patients (31, 71). Vedolizumab could be utilized in cases when infliximab is contraindicated or ineffective (186).

IFX, a TNF-α antagonist, has been verified to promote tumor immunity and relieve resistance to PD-1 inhibitors significantly (195, 197). Few prospective studies are conducted to illustrate the dose of IFX, while different guidelines showed almost universal recommendations that the initial dose is at 5 mg/kg (q2w) until symptoms improve (19, 71, 183, 198). Seventy-two percent of patients recover with one IFX dose, though the requirement for two or three doses is common (19, 77, 196, 197). However, IFX is reported to induce a rare type of hepatitis (199) and is limited in patients with hepatitis B virus or latent tuberculosis history (200, 201). Moreover, IFX utilization is notably correlated with lymphomas and skin cancer in young adults, while this correlation might be confounded by overall increased malignancy risk and previous thiopurine utilization (202). Golimumab, certolizumab, adalimumab, and etanercept could be alternatives to IFX if paradoxical adverse events occur in CIC patients (203).

Vedolizumab, an antagonist of α4β7 on CD4^+^ T cells, prevents T cells from aggregating and homing into the inflammatory intestinal mucosa. The unique gut selective mechanism could avoid systemic immunosuppression without substantially changing antitumor response to chemotherapy (28). It is administered to treat steroid-refractory cases with 300 mg infusions at 0, 2, and 6 weeks or until laboratory or clinical improvement (204, 205). Bergqvist et al. (206) reported seven patients with steroid-refractory or steroid-dependent CIC who recovered by using vedolizumab. CIC with microscopic colitis on histopathology also has a response to vedolizumab (205). The potential risk of tumor progression in patients with lymph nodes metastasis might be minimized by vedolizumab since its gut selectivity (207). In addition, utilizing vedolizumab, which has the same potential as IFX, could not increase secondary tumor risk in vulnerable individuals.

Increasing research studies support the early introduction of biological agents in the therapy algorithm (60, 197, 208). Wang et al. (60) pointed out that a long period of steroid monotherapy could increase the infectious risk in contrast to a short period of steroid such as IFX, which suggests that the early utilization of steroid-sparing therapy might improve the prognosis. Abu-Sbeih et al. (208) recruited 179 CIC patients who received biologic therapy (IFX and vedolizumab) to explore their clinical outcomes. They found that over half of the patients significantly experienced lower hospitalization, shorter symptom duration, shorter steroid taper/taper attempt, and higher success rate of steroid taper. Decreased fecal calprotectin levels and the promoted histologic regression rate were found in patients treated with over three infusions of biologics. Usually, the biologics withdrawal happens in the endoscopy finding of mucosal healing, if there is an attempt to resume ICI therapy. However, no prospective studies have emerged to verify the effectiveness of biologics as first-line therapy. ASCO guidelines stated that individualized therapeutic decisions should be recommended all along (79).

Besides, there are also other potential biologics for refractory ICI-related colitis (203). Anti-IL-1 blockade (anakinra), anti-IL-17 blockade (ixekizumab), anti-IL-23 and anti-IL-12 blockade (ustekinumab) (209), mycophenolate mofetil (MMF) (210, 211), and ciclosporin (54), aiming at blocking critical inflammatory components participating in the pathophysiological process of irAEs, are reported to exert considerable effects in treating severe CIC. Besides, Janus kinase (JAK) inhibitors (tofacitinib) have shown efficacy in CIC patients (212). More studies are warranted to evaluate the usefulness of these biologics in ICI-induced colitis.

### Fecal Transplants

The intestinal microbiome has been hypothesized to play an essential role in the occurrence of CIC. Various gut microbiomes are correlated to induce or alleviate ICI-mediated colitis. Fecal transplantation, contributing to gut microbiome reconstitution and regulatory T cells relatively increasing within the colon mucosa, has been proposed as an optional therapeutic approach for patients with severe or refractory CIC. However, available guidance comes from limited case reports. Wang et al. (213) reported two cases with non-healing CIC who completely recovered after receiving fecal transplantation. Another case that was reported by Fasanello et al. had CIC healed successfully with fecal transplantation (214). Convincing prospective studies are required to verify these observations.

### Surgery

Patients with CIC could deteriorate to colonic perforation, although with the estimated incidence of 1% to 1.5% (71). Emergency colectomy is recommended to be the most effective treatment. Specifically, subtotal colectomy is preferred since the colon lesions are extensive. Segmental colectomy is usually utilized in the remaining severe colonic inflammation in the postoperative period (77). We also included two NSCLC cases with colonic perforation after receiving ICI therapy (50). One recovered after surgery and rechallenge ICIs with ongoing partial regression (50), while the other ultimately succumbed to infectious complications (55). The predictive risk factors for colon perforation are limited. Smith et al. (215) found that CIC patients who received ipilimumab followed with high-dose IL-2 treatment were prone to present perforation than patients who received one of these therapies alone. Heightened vigilance and timely surgery to this severe complication can improve the clinical outcomes effectively.

## Prognosis

Several speculations arise from the association between CIC outcome and survival, whose evidence is rare and conflicting. Two retrospective studies reported that patients who developed ICI-related diarrhea/colitis had a superior OS than those without gastrointestinal toxicity (60, 216). In addition, two simultaneous or sequential irAEs may herald better clinical outcomes. Patients with both colitis and rash caused by immune therapy had a significantly better OS (28.6 vs. 19.9 months, *P* = 0.018) and PFS (16.1 vs. 3.2 months, *P* = 0.001), in contrast to patients with colitis but no rash (217). Obviously, irAEs indicated encouraging tumor response and clinical benefit in patients, which meant longer PFS and OS (216–219). Similarly, our analysis showed that CIC patients with deteriorated or recurrent outcomes had longer OS than those with improved or resolved outcomes (28.5 vs. 13 months, *P* = 0.015) (**Table 4**). Besides, the CIC course was shorter in CIC patients with resolution or improvement, compared with those with deterioration and recurrence (0.84 vs. 6.25 months, *P* < 0.001) (**Table 4**). Moreover, we found that over 60% of patients with grade 2 CIC experienced resolved or improved CIC outcomes, while nearly half of patients with grade 3–4 CIC experienced deteriorated or recurrent CIC outcome (**Table 3**). Generally, higher grade irAEs were reported to suffer inferior prognosis (220). However, several studies showed that no significant correlation was observed between CIC and survival (221, 222). Therefore, the correlation between irAEs and survival needs further verification, and the involved mechanisms are warranted to be clarified.

Predictors for CIC are still on exploration. Several serum markers like CRP, LDH, and fecal calcitonin and their association with CIC prognosis have been investigated. Justine et al. revealed that patients with elevated CRP level had a higher risk of irAE recurrence (223). LDH ≥618 IU/L was related to a poor prognosis in CIC patients (60). Conversely, the decrease of fecal calcitonin after treatment occurred more significantly in patients with CIC remission under endoscopy, which could be used as a non-invasive tool for monitoring and evaluating the response of CIC (159). Nevertheless, the value of fecal calcitonin was limited by its 54% specificity in predicting the endoscopic remission of CIC (166). Furthermore, CIC with immuno-suppressive therapy might be associated with improved OS and PFS compared with CIC without immunosuppressive treatment (25, 60). Higher grade diarrhea could predict great prognosis (216). In addition, the application of antibiotics at any time was reported to decrease the incidence of CIC recrudescence, and patients in our analysis who experienced poor CIC prognosis (*P* = 0.025) or had higher CIC grade (*P* = 0.025) were more frequently utilized antibiotics (**Tables 3** and **4**). However, it could increase the frequency of hospitalization and intensive care unit hospitalization and exacerbate CIC severity (224).

## Rechallenge and Recurrence

A great number of meta-analyses and retrospective studies showed that the resumption after irAEs was potentially effective and relatively safe in selected individuals with careful monitoring (225–229). Universally, patients who improved to grade 1 or less diarrhea/colitis could resume ICI therapy. Any grade 4 irAEs, especially cardiac and neurologic toxicities, are not allowed to continue or reuse ICI (225). The patterns of ICI reintroduction included the same ICI utilized before, the same type of ICIs, and the switching of ICIs of distinct types. Kitagawa et al. (230) showed the safety and efficacy of rechallenging ICIs in patients with NSCLC reported in prior studies. They found that switching ICI class therapy obtained a superior outcome, with over 50% of patients experiencing stable or partial regression disease. Gobbini et al. (231) demonstrated that NSCLC patients discontinuing for irAEs had more prolonged survival than those discontinuing for disease progression. After ICI rechallenge, the recurrence rate of irAEs varied from 37% to 78% (227, 232–234). Among these recurrent irAEs, colitis showed significantly higher incidence than other irAEs (227). Nearly 20%–30% of patients would experience recurrent grade 3 CIC (195, 233). In our analysis, 12.5% (4/32) of patients (36, 43, 50, 54) were retreated with the same ICI therapy used before, one of which experienced the recurrence of grade 3 enterocolitis (54). All of these patients who were reintroduced to ICI therapy harbored deteriorated/recurrent CIC outcomes (*P* = 0.037) (**Table 4**). Furthermore, a multicenter retrospective study compared the clinical outcomes in ICI retreatment and discontinuation group patients (229). The results showed that retreatment patients had fewer steroid requirements and less hospitalizations (*P* = 0.007). Besides, the outcomes of recurrent irAEs were found to be more severe if patients were treated with immunosuppressants before, and thus, they need more intensive immunosuppressive treatment. A retrospective study (205) investigated a patient subset of reinitiation ICI therapy. They found that most of the patients need steroid, while a minority of them required IFX or vedolizumab, regardless of whether in the initial or recurrent episode. Our analysis showed that 25% (8/32) of patients (36, 41, 43, 46, 48, 54, 57) had recurrent ICI-mediated colitis without ICI retreatment, which received subsequent therapy involving probiotics, antidiarrheal drugs, antiviral drugs, steroid, and immunosuppressants. Four cases with CIC finally did not improve or deteriorate after the second treatment (36, 41, 48, 54). The risk factors associated with CIC recurrence included early irAE development, long duration of the initial CIC, initial application of immunosuppressants, pneumonitis, hepatitis, age over 65, advanced tumor stage, CTLA-4 inhibitors use (227), and less antibiotic therapy (205, 224). In addition, the factors—good performance status at ICI resumption, the duration and response of the initial ICI course, longer ICI-free interval, and high PD-L1 expression—were considered so far as correlated to clinical benefit from ICI retreatment (235, 236). Actually, with the increasing interest in immunotherapy reintroduction in recent years, we still need further *ad-hoc*-designed and prospective studies to assess the efficacy and safety of the diverse immunotherapy strategies.

## Summary

ICI-mediated colitis is the most common irAEs. The incidence varied with different types of ICIs, with combined ICI therapy obtaining the highest occurrence and severity. Gut microbiota plays a critical role in the development of CIC. Endoscopic biopsy is the gold standard of diagnosis, and the consideration combined with medication history is necessary. Current management is based on symptomatic and steroid therapy. The utilization of biological agents according to the severity of CIC grade is reasonable and recommended. The association between CIC and survival is obscure and requires to be explored further. Rechallenge of ICIs is on heated debate. On the condition of symptom control, the reintroduction of ICI is worthy of consideration, with persistent monitoring and evaluation certainly.

## Strengths and Limitations

This review summarized the latest studies to demonstrate the comprehensive characteristics of ICI-related colitis, especially the mechanisms and treatment. We also collected the CIC cases with NSCLC as an example to analyze the differences between different CIC grades and outcomes. The association between endoscopic findings and the severity of ICIs-related colitis has been evaluated as well. We hoped that our comprehensive review could bring some inspiration in clinical practice. In addition, we explored the correlation between initial steroid dose and CIC outcome, but no significant results were found due to the small sample size and limited accurate data. Although we focused on the NSCLC cases with ICI-mediated colitis, the understanding of irAEs is regardless of cancer species. Moreover, we are conducting real-world studies in our center and we hope that large sample data could present more reliable results.

## Future Direction

In the future, early prediction and detection and timely and effective treatment are the two main aspects we need to investigate continuously.

Most studies have focused on the predictive factors for survival outcomes or treatment response (237, 238), while comparatively limited research studies could find biomarkers characterizing irAEs (239, 240). Lim et al. established the CYTOX score covering 11 proinflammatory cytokines and validated its efficacy in predicting severe irAEs which need high-dose corticosteroid therapy (241). However, in view of the extensive organ specificity or systemic presentations in irAEs and the distinct potentially inflammatory mechanisms concerning the adverse events, it is still difficult to translate relevant results to clinical practice. Besides, specific biomarkers are warranted to be explored with a large number of verified preclinical models.

As for the treatment, the optimal steroid dose and continuous/taper time remain inconclusive. Besides, steroid utilization for treating irAEs has become a field with growing controversy. Quite a bit of evidence suggested that the antitumor efficacy of ICI could be weakened by the wide range of immunosuppressive function of steroid (242–244). However, a single-center study (245) recruited 372 patients according to the different indications (irAEs and others) for steroid. They found that using systemic steroid for irAEs could not influence tumor response and OS, while for other indications (usually correlated to poor prognosis), it could. In addition, we also know little about the immune microenvironment effect by systemic corticosteroids administration. A few case reports implied a temporal correlation between steroid therapy, decreased cytokine expression, and irAE resolution (96). Notably, Tyan et al. (143) found that steroid could disrupt the specific harmonization pattern in irAE patients, which suggested the elimination of activated immune states and thus verified the worse outcome in patients without irAEs or in patients treated with steroid. More subgroup analysis is required to associate irAEs and steroid utilization with survival. Except for steroid treatment, current clinical trials, concerning the use of IFX and vedolizumab (NCT04407247) for CIC patients and tofacitinib (NCT04768504) for refractory CIC patients, are looking forward to presenting profound results. Clinical studies on fecal transplant involved in the gut microbiome have also been in the phase of recruiting participants (NCT03819296 and NCT04038619).

Numerous scientists have been persistently working to investigate the development of irAEs. We hope that one day in the future, irAEs could be specifically predicted and its risk could be assessed by simple and convenient detection biomarkers. The cooperation among multiple disciplines should be strengthened for the continuous monitoring of CIC. Novel treatment strategies are expected with a few side effects. Furthermore, we also need more research studies about the clinicopathological and biological characteristics of CIC to help us better understand this specific subset of irAEs.

## Statistical Analysis

We analyzed the differences of CIC onset in different ICI therapy groups by Mann–Whitney *U* test. The descriptive analysis was conducted to describe the baseline features and the intergroup discrepancies in distinct CIC grade and CIC outcome groups. Fisher’s exact or chi-square test and Kruskal–Wallis test were performed to analyze categorical and continuous variables, respectively. Categorical variables were expressed as counts and proportions. Continuous variables were delineated as means and standard deviations. We utilized the statistical software packages R and EmpowerStats (X&Y Solutions Inc., Boston, MA, USA) to perform all the statistical analyses. The statistical significance was presented as two-sided *P*-values less than 0.05.

## Author Contributions

LT and JW searched the literature and wrote the manuscript. NL and YZ helped collect the literature and participated in the discussion. LT and WH performed the statistical analysis. JL and XM examined and verified the study. All authors contributed to the article and approved the submitted version.

## Conflict of Interest

The authors declare that the research was conducted in the absence of any commercial or financial relationships that could be construed as a potential conflict of interest.

## Publisher’s Note

All claims expressed in this article are solely those of the authors and do not necessarily represent those of their affiliated organizations, or those of the publisher, the editors and the reviewers. Any product that may be evaluated in this article, or claim that may be made by its manufacturer, is not guaranteed or endorsed by the publisher.
